# Exploring behavior change motivation in an outpatient sample with more than one health risk behavior

**DOI:** 10.1093/eurpub/ckac131.365

**Published:** 2022-10-25

**Authors:** L Voigt, A Ullrich, S Ulbricht

**Affiliations:** 1 Prevention Research and Social Medicine, University Medicine Greifswald, Institute for Community Medicine, Greifswald, Germany; 2 Partner site Greifswald, German Centre for Cardiovascular Research, Greifswald, Germany

## Abstract

**Background:**

Evidence from western countries shows that the majority of adults have two or more health risk behaviors. The motivation to engage in a health behavior change (HBC) is the most proximal determinant of behavior change in the future. The aim of this study was to investigate the intention to increase physical activity and to quit smoking in an outpatient sample that show both health risk behaviors.

**Methods:**

We used baseline data (n = 109) of an intervention study (Germany, 2016-2019) on physical activity and smoking cessation. Eligibility criteria were: aged 40 to 65 years, systolic blood pressure ≥130 mmHg, no history of cardiovascular event or vascular intervention. We collected information on HBC motivation, sex, age, and self-rated health (SRH) and identified 32 physically inactive smokers. Descriptive analyses and Fisher’s exact test were used to explore the proportion of those motivated for HBC and differences according to sex, age (45-55 vs. 56-65 years), and SRH (excellent-good vs. poor-very poor).

**Results:**

Participants (50% female) were on average 52.6 years old and 78% had school education ≤10 years. Overall, 44% intended to change physical inactivity only, 34% intended to change both behaviors, 13% wanted to change neither, and 9% intended to change smoking only. There was no difference in HBC motivation according to sex and age. But, there was a significant difference according to SRH (Fisher’s exact = 0.048); e.g., there were more individuals motivated to change both behaviors among those with lower SRH (64%) compared to those with higher SRH (19%).

**Conclusions:**

In this baseline sample of an intervention study, the majority of participants intended to change physical inactivity but not smoking. Among those with lower SRH, almost two-thirds intended to change both behaviors. Thus, individuals with more than one health risk behavior differ in their HBC motivation. Lower SRH may offer a window of opportunity to promote HBC interventions.

**Key messages:**

• Physically inactive smokers with a systolic blood pressure ≥130 mmHg differ in their motivation to change these behaviors.

• Lower self-rated health may offer a window of opportunity to promote interventions to change health behavior.

